# eIF3a Regulates Colorectal Cancer Metastasis *via* Translational Activation of RhoA and Cdc42

**DOI:** 10.3389/fcell.2022.794329

**Published:** 2022-03-01

**Authors:** Chao Mei, Chong Liu, Ying Gao, Wen-Ting Dai, Wei Zhang, Xi Li, Zhao-Qian Liu

**Affiliations:** ^1^ Department of Clinical Pharmacology, Hunan Key Laboratory of Pharmacogenetics, National Clinical Research Center for Geriatric Disorders, Xiangya Hospital, Central South University, Changsha, China; ^2^ Institute of Clinical Pharmacology, Engineering Research Center for Applied Technology of Pharmacogenomics of Ministry of Education, Central South University, Changsha, China

**Keywords:** eIF3a, Rho GTPases, pseudopodia, metastasis, cytoskeleton

## Abstract

Tumor metastasis is the major cause of tumor relapse and cancer-associated mortality in colorectal cancer, leading to poor therapeutic responses and reduced survival. eIF3a was previously described as an oncogene. However, its role in colorectal cancer progression and metastasis has not yet been fully investigated. In this study, the expression specificity and predictive value of eIF3a were investigated in clinical samples. The effects of eIF3a on cell proliferation and migration were verified *in vivo* and *in vitro*, respectively. The underlying molecular mechanism was revealed by western blotting, immunofluorescence, RNA-binding protein immunoprecipitation, and dual-luciferase reporter gene assays. The results showed that eIF3a was significantly overexpressed in tumor tissues compared with adjacent normal tissues. High eIF3a expression was correlated with tumor metastasis and overall survival. Downregulation of eIF3a obviously inhibited the proliferation and motility of malignant cells *in vitro* and *in vivo*. Mechanistically, eIF3a regulates Cdc42 and RhoA expression at the translation level, which further affects pseudopodia formation and actin cytoskeleton remodeling. Taken together, eIF3a accelerates the acquisition of the migratory phenotype of cancer cells by activating Cdc42 and RhoA expression at the translational level. Our study identified eIF3a as a promising target for inhibiting colorectal cancer metastasis.

## Introduction

Colorectal cancer is the third most common cancer type with increasing incidence and mortality (Dekker et al., 2019; Keum and Giovannucci, 2019). The majority of mortalities are characterized by metastasis, which is the spread of primary tumors to colonize distant locations. The mechanisms underlying metastasis, however, remain poorly understood (Allgayer et al., 2020). For metastasis to occur, malignant cells must first undergo coordinated processes to acquire a premigratory phenotype (Mierke, 2019; Clayton and Ridley, 2020; Guan et al., 2020). These processes include cytoskeletal reorganization, cell adhesion alteration, invasive structure formation, and surrounding stromal degradation (Tang et al., 2018; Kai et al., 2019; Lauko et al., 2020; Li and Wang, 2020). Such cytoskeletal dynamics and intracellular signaling are mainly regulated by the Rho family of small guanosine triphosphatases (GTPases) (Lawson and Ridley, 2018; Svensmark and Brakebusch, 2019). Rho family GTPases comprise over 60 members (Jansen et al., 2018). One of the most characterized enzymes, cell division cycle 42 (Cdc42), plays an irreplaceable role in cell polarization, filopodia formation, cytoskeletal reorganization, cell proliferation, and migration (Stengel and Zheng, 2011). Another member of the Rho family, ras homologue family member A (RhoA), is hyperactivated in several types of human cancer (Jansen et al., 2018; Nam et al., 2019). RhoA primarily mediates actin stress fiber formation and contractility (Narumiya et al., 2009). When activated, RhoA switches to an activated GTP-bound form and transmits the signal to downstream effectors, including the Rho-associated serine-threonine kinase (Rock), promoting actin cytoskeleton reorganization and a variety of cellular functions (Narumiya et al., 2009; Wei et al., 2016).

Eukaryotic translation initiation factor 3a (eIF3a) is the largest subunit of eIF3 (Yin et al., 2018). eIF3a is widely recognized as a proto-oncogene correlated with tumor prognosis, occurrence, metastasis, and therapeutic response (Xu et al., 2017; Luo et al., 2018; Chen et al., 2021). Accumulating studies have reported eIF3a overexpression in several types of cancers. Ectopic upregulation of eIF3a promotes cell proliferation and malignant transformation (Zhang et al., 2007), whereas knockdown of eIF3a inhibits cell growth, migration, invasion, and colony formation in bladder, lung, and pancreatic tumor cells (Dong et al., 2004; Spilka et al., 2014; Wang et al., 2016). In addition, eIF3a also participates in many cellular processes, including cell cycle, differentiation, DNA synthesis, DNA repair, fibrosis, and chemotherapy resistance (Dong et al., 2009; Spilka et al., 2012; Xu et al., 2012; Chen et al., 2021). With the increasing understanding of the physiological and pathological functions of eIF3a, it is gradually considered as a new potential anticancer target. However, its specific role in colorectal cancer and the detailed mechanism of tumor metastases have not yet been investigated (Wang et al., 2016).

This study characterized the role of eIF3a in colorectal cancer metastasis. We found that eIF3a is selectively overexpressed in tumor tissues and positively correlated with the metastatic phenotype and poor prognosis in colorectal cancer patients. In addition, we revealed a novel role of eIF3a in tumor acquisition of invasive properties, including abundant pseudopodia formation and cytoskeleton reconstruction, by translationally regulating the expression of Cdc42 and RhoA. These findings suggested that eIF3a may serve as a promising prognostic marker and therapeutic target for mitigating lethal metastasis in colorectal cancer patients.

## Materials and Methods

### Patients and Clinical Specimens

From 2016 to 2021, 140 fresh colorectal tumor and adjacent normal samples were acquired from colorectal cancer patients who underwent surgery in Xiangya Hospital. None of the patients had received any anticancer therapies prior to surgery. On the basis of the World Medical Association International Medical Ethics, patients involved in this research provided written informed consent before surgery. This research was approved by the Independent Ethical Committee of Institute of Clinical Pharmacology, Central South University.

### Cell Lines and Culture Conditions

The human colorectal cancer cell lines HCT116 and CACO2 were maintained in Gibco Roswell Park Memorial Institute (RPMI) 1640 medium. HT29 cell line was cultured in McCOY’s 5A. The human embryonic kidney 293T cell line was maintained in DMEM. All media were supplemented with 10% fetal bovine serum (FBS) (Gibco), and cells were cultured in an incubator at 37°C in a humid atmosphere containing 5% CO_2_.

### Small Interfering RNA and Plasmid Transfection

Human siRNAs for eIF3a were purchased from RiboBio (Guangzhou, China) and used with RNAimax (Invitrogen) reagent following the manufacturer’s protocol. For the overexpression assay, overexpression vector and control empty plasmid were purchased from Gene (Shanghai, China). Transfection with plasmids was performed with Lipofectamine 3000 (Invitrogen) reagent following the manufacturer’s instructions. The siRNA sequences used in this study are listed in Supplementary Table S1.

### Cell Viability Assay

Cells were plated in 96-well plates (3 × 10^3^ per well). Cell viability was measured at the indicated times using Cell Counting Kit-8 (CCK-8, Bimake, Shanghai, China) reagents. CCK8 was mixed in culture medium at a ratio of 1:9 and then added to each well (100 μl per well). One hour later, the absorbance was read at 450-nm wavelength at a ratio of 1:9 using a spectrophotometer. Each group included six replicate wells.

### Colony Formation Assay

Cells were resuspended in a single-cell suspension and seeded in six-well culture plates (1,000 cells per well). Then, the cells were left to grow for 2 weeks. At the end of incubation, cells were fixed with 4% paraformaldehyde for 20 min, stained with crystal violet for 30 min, and washed with PBS. The colonies were photographed and counted from three independent experiments.

### Wound Healing Assay

Cells were seeded into six-well plates and cultured in a cell incubator to form a confluent monolayer. Then, the cells were streaked with a sterile pipette tip, washed twice with PBS to remove cell debris, and supplemented with new low-serum medium (containing 0.5% FBS). Images were captured under a microscope at 0, 24, and 48 h after wounding. Wound widths were measured, and the average rate of wound closure was calculated as the percentage of closure wound distance in relation to the initial wound distance.

### Transwell Penetration Assay

Inserts were placed in a 24-well culture plate. Complete medium (500 μl, containing 10% FBS) was added to the bottom of the wells. Cells were resuspended in serum-free medium and then seeded into the upper chamber at a concentration of 1 × 10^4^ per well. Forty-eight hours later, the cells remaining in the upper chambers were wiped with cotton swabs. Then, 4% paraformaldehyde was used to fix the cells that migrated to the bottom surface of the membrane for 20 min. Then, the cells were stained with crystal violet for 30 min and washed with PBS. Migrated cells were photographed and counted under a microscope.

### Quantitative Real-Time PCR

Total RNA was extracted using RNAiso reagent (Takara, Japan). RNA was transcribed into cDNA with the PrimeScript™ RT Reagent Kit (Perfect Real Time) (Takara, Japan) following the instructions. Real-time PCR was performed with SYBR Premix Ex Tap™ (Takara, Japan). Values for each gene were normalized to GAPDH. The relative expression of targeted genes was calculated using the 2^−ΔΔCt^ method. The primers for candidate genes are listed in Supplementary Table S2.

### Animal Experiments

For the subcutaneous tumor formation assay, 12 male BALB/c nude mice (5–6 weeks old) were randomly divided into two groups (*n* = 6). A xenograft model of colorectal cancer was established by subcutaneous injection of 5 × 10^6^ HCT116 cells with stable eIF3a knockdown or negative control. The shRNA sequences are listed in Supplementary Table S3. Tumor minimum (*W*) and maximum (*L*) diameters were measured every 3 days. Tumor volumes were calculated using the following formula: *V* = 1/2 × length × width^2^. All mice were sacrificed 3 weeks after injection.

To establish a metastasis model, 12 BALB/c-nude mice (male, 5–6 weeks old) were randomly divided into two groups (*n* = 6/group). To examine the metastasis ability, 2 × 10^6^ HCT116 cells were intravenously injected into mice. Mice were euthanized 2 months later. Lungs and livers were surgically isolated, and metastatic lesions were observed.

The animal studies were conducted with the approval of the Animal Ethics Committee of the Third Xiangya Hospital of Central South University, and all possible methods were performed to minimize the suffering of animals.

### Immunohistochemistry

Immunohistochemistry (IHC) staining for paraffin-embedded human colorectal tumor and adjacent normal tissue sections was performed using anti-eIF3a antibody (ab128996, Abcam, United States) following the manufacturer’s protocol. Antibodies against eIF3a (ab128996, Abcam, United States) and Ki67 (ab16667, Abcam, United States) were used for IHC analysis of mouse tumor sections. The staining intensity was analyzed and scored (ranging from 0 to 9) according to the intensity multiplied by the extent of stained cells.

### Western Blot Analysis

Cells were lysed in ice-cold RIPA lysis buffer containing protease inhibitor (Beyotime, China) and incubated on ice for 30 min. Then, cell lysates were centrifuged, and the supernatants were transferred to a new tube for protein concentration determination (BCA assay, Beyotime Biotechnology, China). A 5× loading buffer was added to the sample to obtain a 1× final solution. The samples were then heated at 95°C–100°C for 10 min and cooled to room temperature before separation by SDS-PAGE. Proteins were separated on gels, transferred to PVDF membranes (Merck Millipore), and blocked in 5% skim milk at room temperature. The membranes were first incubated in primary antibodies at 4°C overnight and then in secondary antibodies at room temperature for 1 h. The signal was visualized with enhanced chemiluminescence (Invitrogen, Carlsbad, CA, United States). The relative expression was quantified using Image Lab 6.0 and normalized to blots of negative control (NC). The antibodies used were as follows: eIF3a (Abcam, ab128996); Gapdh (Sigma, SAB1405848); Cdc42 (Proteintech, 10155-1-AP); Rhoa (Proteintech, 10749-1-AP); ROCK1 (WL01761); ROCK2 (WL00500); and cortactin (ab81208).

### Immunofluorescence

Cells were seeded on glass coverslips. After specific treatment, cells were rinsed thrice in PBS, fixed with 4% paraformaldehyde for 20 min, permeabilized with 0.5% Triton X-100 for 10 min, and then blocked in 1% BSA for 1 h at room temperature. Then, the samples were incubated overnight with primary antibodies at 4°C and then incubated with secondary antibodies and phalloidin at room temperature for 1 h. Slides were prepared using DAPI and observed with a fluorescence microscope. Antibodies against Rhoa (Proteintech, 10749-1-AP) and cortactin (Abcam, ab81208) were used for this analysis. Phalloidin was obtained from Abcam (ab176753).

### Luciferase Reporter Assay

For this assay, HCT116 and HT29 cells were plated with eIF3a or NC siRNA for 24 h. Then, cells were plated into 24-well cell plates and cotransfected with luciferase plasmids along with Renilla luciferase vector as a transfection efficiency control using Lipofectamine 3000. The Dual Luciferase Reporter Assay Kit (Promega) was used to test luciferase activity 48 h after transfection. Cells were first lysed in 100 μl 1× passive lysis buffer and vortexed for 15 min. Then, 100 μl LARII reagent was added to 20-μl cell suspensions for each assay followed by firefly luciferase activity testing. Then, 100 μl Stop & GloR reagent was added to the tube, and the Renilla luciferase activity was tested. Firefly luciferase activity was normalized to the Renilla luciferase signal.

### RNA Immunoprecipitation

The RNA immunoprecipitation (RIP) experiment was performed using an EZMagna RNA Immunoprecipitation Kit (Millipore, Billerica, MA) following the manufacturer’s instructions. Briefly, 293T cells were lysed and incubated with anti-IgG or anti-eIF3a antibodies and lightly rotated at 4°C overnight. The purified RNA was analyzed by quantitative real-time PCR.

### Statistical Analysis

All results were calculated from three independent experiments. The correlation analyses were assessed by Pearson’s correlation test. The Kaplan–Meier survival curve was generated using a log-rank test. Statistical analyses were assessed by paired or unpaired two-tailed Student’s *t* tests or the Mann–Whitney *U* test in GraphPad Prism 5 (GraphPad, San Diego, CA, United States). A *p* value <0.05 was considered statistically significant (*p < 0.05, **p < 0.01, ***p < 0.001, and ****p < 0.0001).

## Results

### eIF3a is Overexpressed in Colorectal Tumors and Correlated With Progressive Diseases

**FIGURE 1:**
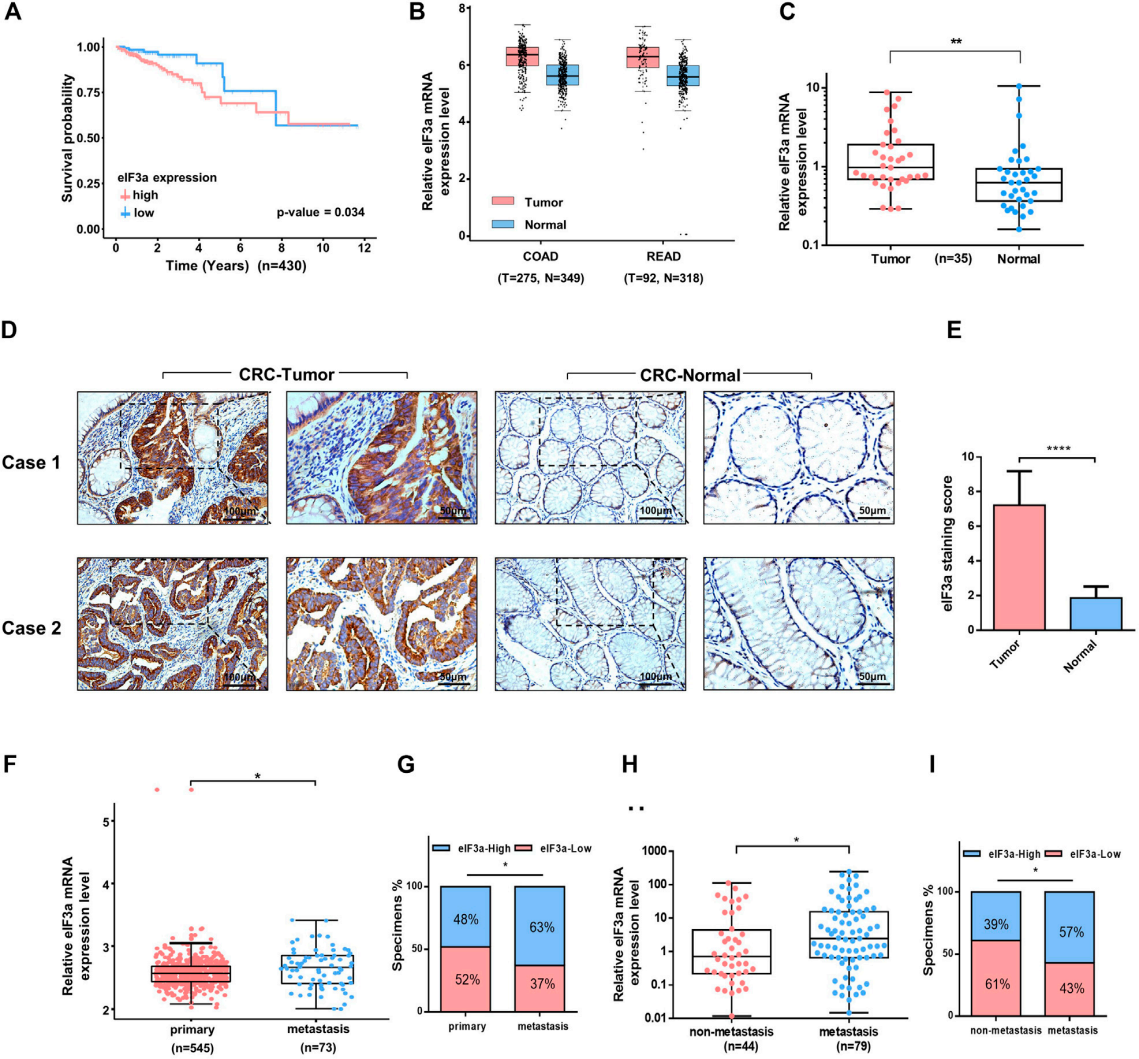
Clinical features of eIF3a in colorectal cancer. **(A)** A total of 430 colorectal tumor samples obtained from The Cancer Genome Atlas (TCGA) colorectal cancer dataset were included in the survival analysis. **(B)** eIF3a was significantly overexpressed in colon (COAD) and rectal tumors (READ) compared to normal tissues. This analysis was performed in GEPIA. **(C)** The expression of eIF3a mRNA was evaluated in 35 pairs of colorectal tumor and adjacent normal tissues by q-PCR assay. **(D)** IHC staining of eIF3a was performed in sections of colorectal tumor and adjacent normal tissues. **(E)** Statistical result of IHC staining scores. **(F)** The mRNA expression of eIF3a was analyzed in the primary tumor tissues of colorectal cancer patients with metastatic tissues based on expression files obtained from the Gene Expression Omnibus (GEO) datasets (GSE131418). **(G)** The percentage of colorectal cancer patients with high (blue) and low (red) eIF3a expression was reported for metastasis tissues (right) and primary tumor tissues (left). Metastatic tissues were more likely to have a relatively higher eIF3a expression level. **(H)** eIF3a mRNA expression was analyzed in clinical colorectal tumor samples with or without metastatic disease. **(I)** The percentage of patients with different levels of eIF3a expression (blue—high; red—low) with (right) and without (left) metastatic disease. COAD, colon cancer; READ, rectal cancer; CRC, colorectal cancer.

eIF3a exhibits oncogenic behavior in several types of cancer; however, its role in colorectal cancer remains unclear. To determine the potential function of eIF3a in colorectal cancer, we first analyzed the effect of eIF3a expression on the overall survival of colorectal patients in The Cancer Genome Atlas (TCGA) datasets. Interestingly, patients with higher eIF3a expression presented poorer survival outcomes, whereas patients with lower eIF3a expression exhibited a better prognosis (Figure 1A) We then evaluated eIF3a expression in colorectal tumor tissues versus normal tissues. By examining GEPIA (http://gepia.cancer-pku.cn/index.html), we found that eIF3a mRNA expression was significantly increased in tumor tissues, both in colon cancer and in rectal cancer (Figure 1B). These results were further confirmed in our own clinical samples (Figure 1C). Additionally, using immunohistochemistry assays, we observed an increase in eIF3a protein levels in tumor tissues compared to adjacent normal tissues (Figures 1D,E). The above data consistently verified the abnormal expression of eIF3a in colorectal tumors, which prompted us to further investigate its potential effects on tumor progression and metastasis. By analyzing clinical samples, we found that eIF3a expression in the metastasis sites is higher than primary tumors (Figures 1F,G). Besides, patients with tumor metastasis exhibited significantly higher eIF3a expression than those with non-metastatic disease, demonstrating that approximately 60% of metastatic patients expressed moderate or strong eIF3a. In contrast, non-metastatic patients mostly exhibited low or negative eIF3a expression (Figures 1H,I). In summary, the above results confirmed the abnormal expression of eIF3a in colorectal cancer. A higher eIF3a expression also indicated the tendency of metastatic diseases and poor prognosis, as typically noted for tumor promoters.

### eIF3a Is Necessary for Tumor Metastasis

**FIGURE 2:**
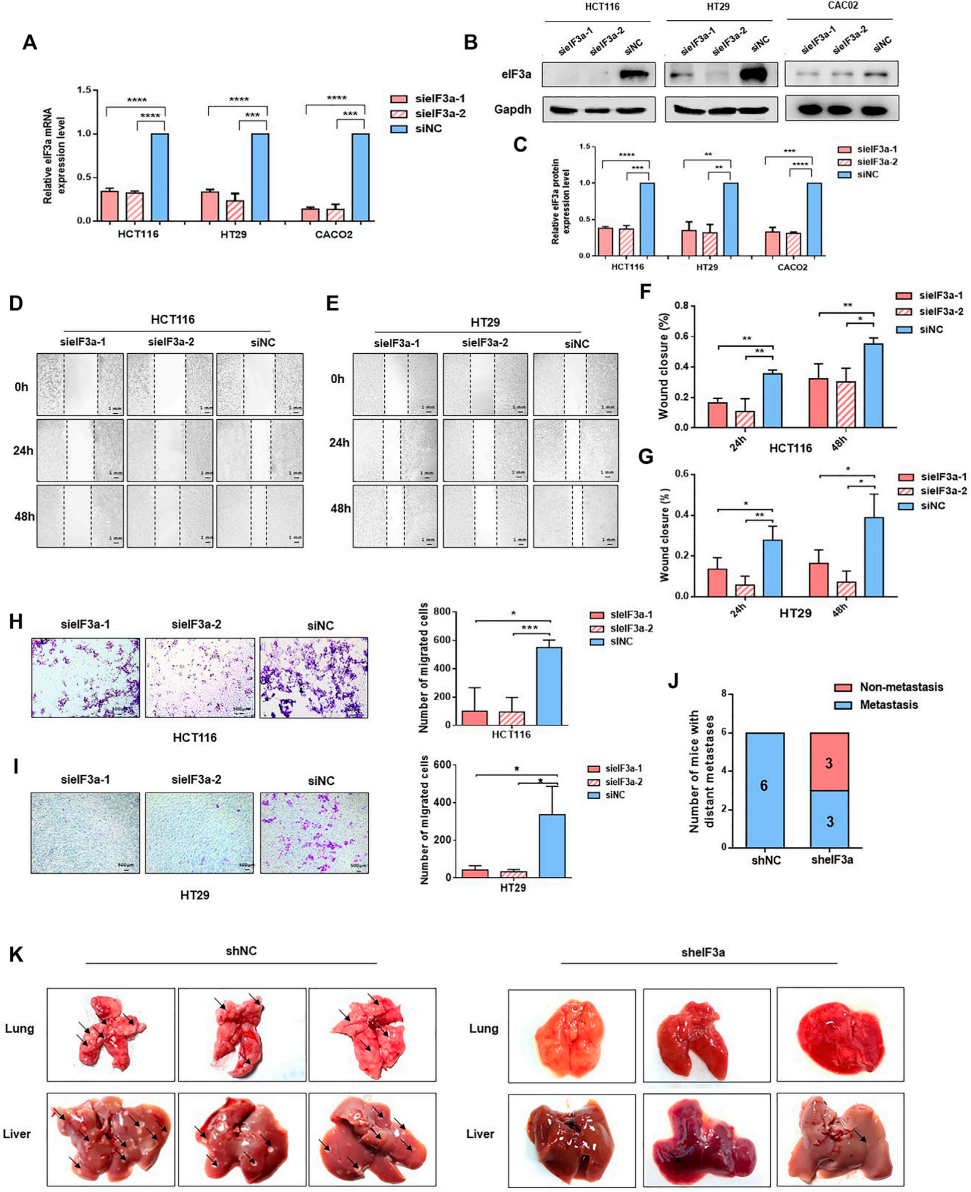
eIF3a regulates tumor metastasis *in vitro* and *in vivo*. **(A)** eIF3a was knocked down with two specific siRNAs in HT29, Caco2, and HCT116 cell lines. q-PCR analysis was performed to verify the knockdown efficiency at the mRNA level. **(B)** Western blot analysis was used to verify the eIF3a knockdown efficiency at the protein level. **(C)** The statistical results of western blot assays. **(D–G)** Representative images **(D,E)** and quantification **(F,G)** of wound healing assays. Images were captured at 0, 24, and 48 h. **(H,I)**. Transwell penetration assay. Typical images and quantification of invading HCT116 **(H)** and HT29 **(I)** cells are presented. **(J)** HCT116 cells (1 × 10^6^) were injected into the caudal vein of nude mice (*n* = 6/group). Mice were sacrificed 2 months after injection. Metastatic lesions in the lung and liver were observed, and mice with or without metastatic nodes were evaluated. **(K)** Representative images of lung and liver tissue are shown. sieIF3a, small interfering RNA targeting eIF3a; siNC, control small interfering RNA; sheIF3a, short harpin RNA targeting eIF3a; shNC, control short harpin RNA.

In consideration of the potential accelerative effect of eIF3a in the metastasis of colorectal cancer patients indicated before (Figures 1F–I), the biological functions of eIF3a on malignant cell metastasis were investigated. We depleted eIF3a in colorectal cancer cells and then performed cell wound healing and Transwell migration assays. The eIF3a-silencing efficiency was verified by q-PCR and western blot assays (Figures 2A–C). For the cell wound healing assay, silencing eIF3a robustly impaired the mobility of tumor cells, as indicated by the relative wound closure width (Figures 2D–G). The Transwell migration assay consistently indicated that eIF3a knockdown remarkably suppressed the cell migration ability (Figure 2H,I). The effects of eIF3a overexpression on cellular proliferation and migration were also investigated (Supplementary Figure S1). Taken together, these results demonstrated that eIF3a contributes to colorectal cancer cell metastasis and motility *in vitro*.

To better simulate the internal environment of colorectal cancer patients, the role of eIF3a in tumor metastasis was investigated in lung colonization mouse models. HCT116-sheIF3a and shNC cells (2 × 10^6^) were injected into different groups of BALB/c nude mice *via* the caudal vein. Mice were euthanized 2 months after inoculation. Then, lung and liver tissues were subjected and analyzed. As shown in Figure 2J, all six mice in the control group underwent serious tumor metastasis, whereas only half of the mice in the eIF3a knockdown group developed distant metastasis. More specifically, silencing eIF3a remarkably reduced the number of tumor metastasis nodules formed in the lung and liver (Figure 2K). These results revealed that eIF3a indeed played a vital role in the metastasis of colorectal cancer cells *in vitro* and *in vivo*.

### eIF3a Suppression Inhibits the Growth of Colorectal Cancer Cells

**FIGURE 3:**
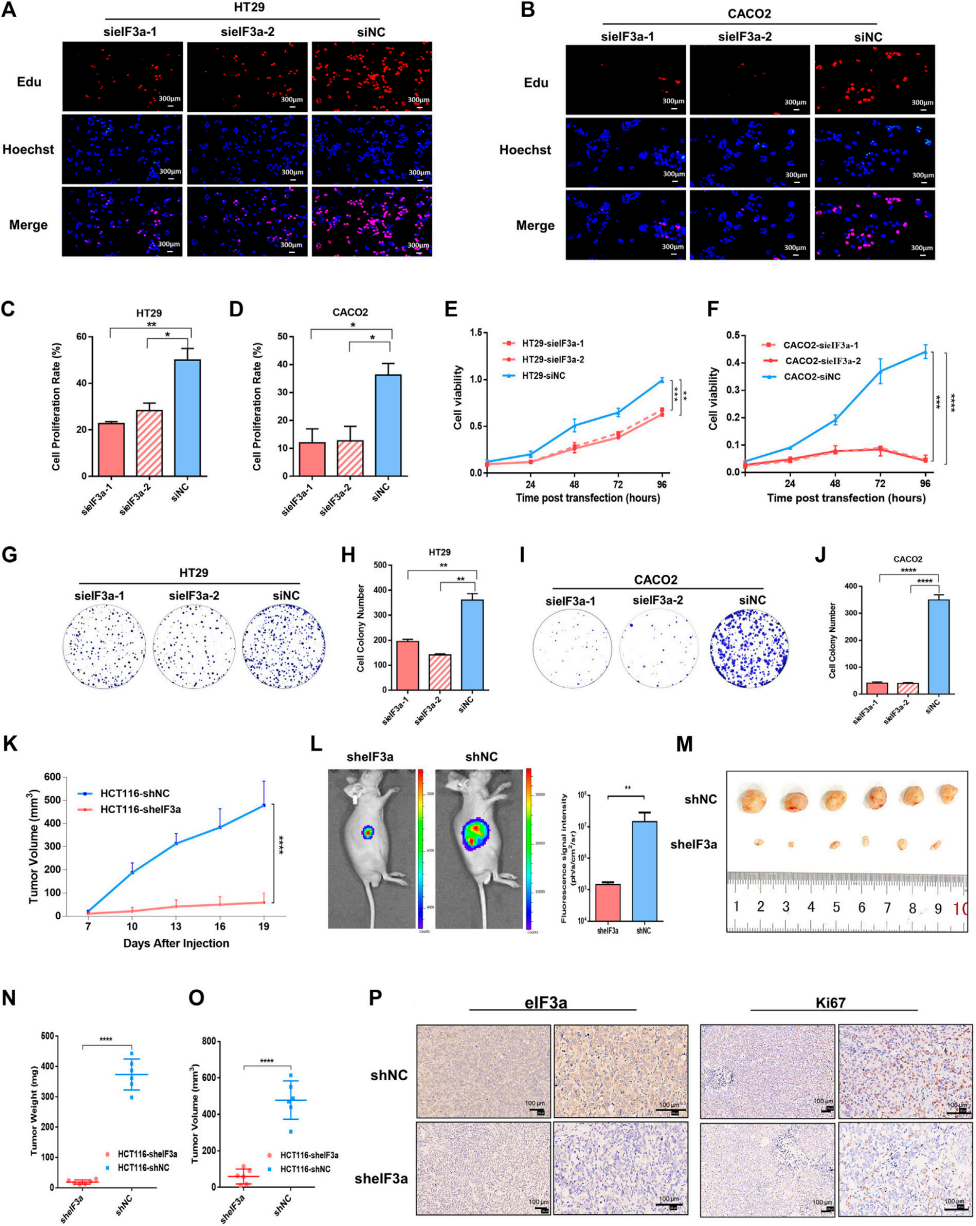
eIF3a regulates tumor proliferation *in vitro* and *in vivo.* **(A,B)** EdU assays were performed to evaluate the alterations in cell proliferation ability after eIF3a suppression. **(C,D)** The statistical results of EdU assays, which were calculated from three independent experiments. **(E,F)** Cell viability was analyzed by CCK-8 assay. **(G–J)** Clone formation analyses. Statistical results were calculated from three independent experiments. **(K)** Tumor growth and volume were measured every 3 days. **(L)** Tumor growth status was monitored *via in vivo* imaging. **(M)** Isolated xenograft tumors from different groups of nude mice. **(N,O)** The volumes and weights were measured for each tumor. **(P)** Representative images of eIF3A (left) and Ki67 (right) detected by IHC analysis. sieIF3a, small interfering RNA targeting eIF3a; siNC, control small interfering RNA; sheIF3a, short harpin RNA targeting eIF3a; shNC, control short harpin RNA; EdU, 5-ethynyl-2′-deoxyuridine.

To mechanically investigate the role of eIF3a in tumor progression, we first examined the impact of eIF3a knockdown on tumor cell proliferation by performing an EdU staining assay. The percentage of EdU-stained cells demonstrated that compared with control cells, eIF3a downregulation noticeably decreased cell proliferation (Figures 3A–D). The CCK-8 cell viability assay was also performed, and the cell proliferation rate was significantly inhibited after eIF3a suppression (Figures 3E,F). Consistently, the colony formation assay also revealed a positive regulatory role of eIF3a in tumor proliferation ability (Figures 3G–J). Mechanistically, eIF3a inhibits and upregulates the expression of p27^kip1^ and ribonucleotide reductase M2 (RRM2), respectively, both of which play critical roles in cell proliferation and cell cycle regulation. The results are consistent with previous studies (Supplementary Figure S2) (Dong and Zhang, 2003; Dong et al., 2004; Dong et al., 2009). In conclusion, eIF3a plays a vital role in colorectal cancer cell proliferation and viability, which also partly explains the aberrant expression of eIF3a in colorectal tumor samples.

The role of eIF3a in malignant cell proliferation and viability was further studied *in vivo* by constructing a nude mouse xenograft model. A total of 12 nude mice were randomly divided into two groups. Then, eIF3a stable knockdown and control HCT116 cells were transplanted into different groups *via* subcutaneous injection. Tumor volume was monitored every 3 days (Figure 3K). An *in vivo* bioluminescence imaging assay was performed to monitor the growth situation of the subcutaneous transplantation (Figure 3L). Obviously, the tumor growth rate was notably inhibited by eIF3a knockdown (Figure 3M). All nude mice were euthanized after 3 weeks, and tumors were subjected to further investigation. The volumes and weights of each tumor were measured and calculated. As shown in the results, HCT116-sheIF3a tumors were significantly smaller and lighter than control tumors (Figures 3N,O). In addition, the expression of Ki67, a classical and dependable marker of the cell proliferation rate, was evaluated by immunohistochemical assays. Ki67 expression exhibited an obvious decrease after eIF3a knockdown (Figure 3P). Collectively, these results further demonstrated the oncogenic role of eIF3a in colorectal tumor growth *in vivo*.

### eIF3a is Required for Pseudopodia Formation and Cytoskeleton Reorganization

**FIGURE 4:**
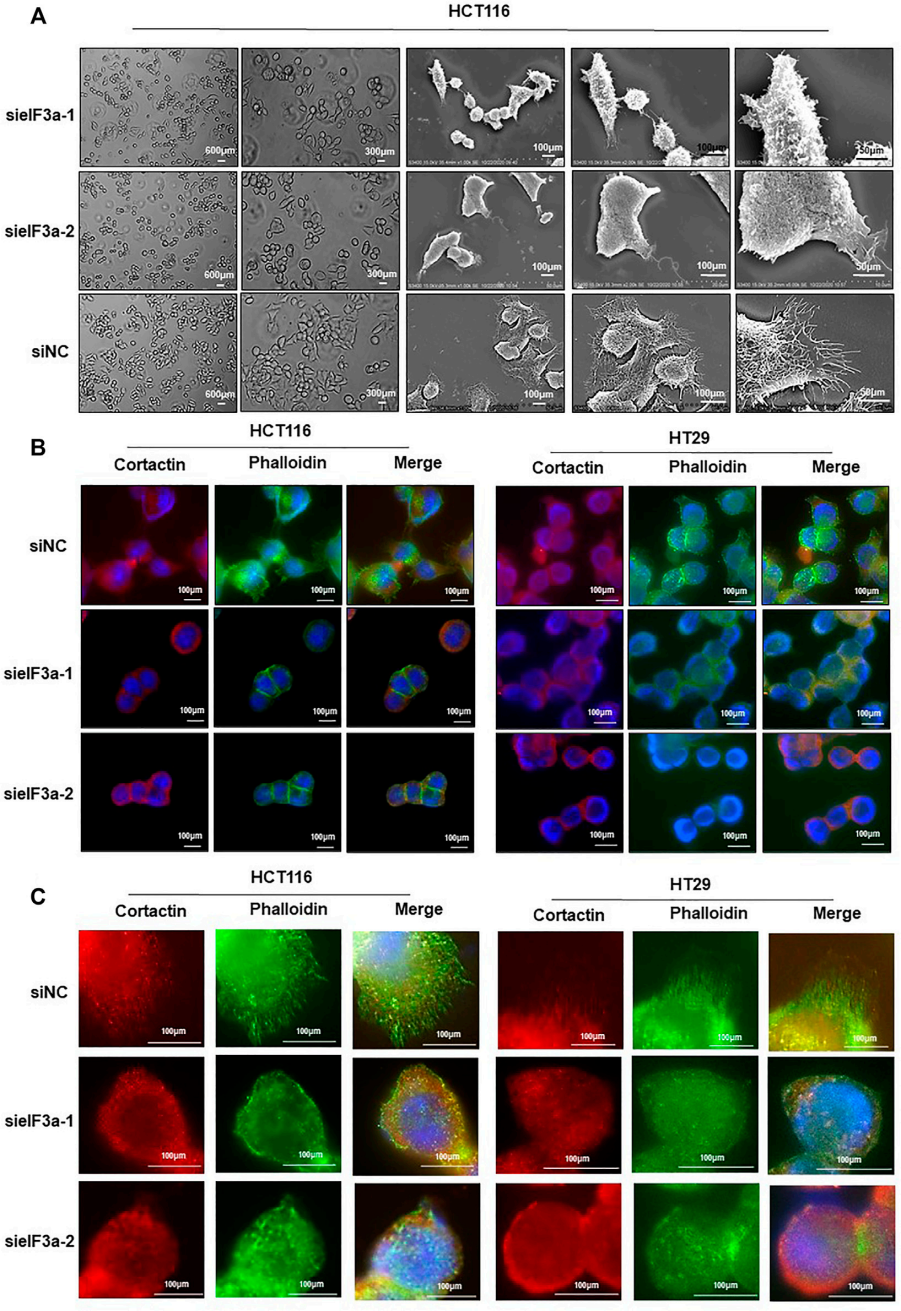
Downregulation of eIF3a affects pseudopodia formation. **(A)** Optical microscopy and electron microscopy were used to assess HCT116 cellular morphology after eIF3a knockdown at different magnification times. **(B,C)** F-actin stained with FITC-phalloidin (green) and cortactin (red) was visualized in control and eIF3a-silenced HCT116 and HT29 cells. sieIF3a, small interfering RNA targeting eIF3a; siNC, control small interfering RNA.

eIF3a knockdown significantly inhibited cell motility, as verified above, prompting us to assess the molecular mechanism of cell motility modulation. Malignant cells maintain continuous forward movement of the leading edge of the plasma membrane by reorganizing the cytoskeleton to form pseudopodia, specialized actin-rich membrane structures that largely form in invasive cancer cells (Jacquemet et al., 2015). To test this possibility, we monitored the cellular morphology alterations at different magnifications in response to eIF3a knockdown. As shown in Figure 4A, control cells presented more spike-like morphology with abundant membrane protrusions, whereas eIF3a-depleted cells exhibited less spread with a significant decrease in membrane protrusions. We also tested proteins enriched in pseudopodia by detecting their expression and distribution under a fluorescence microscope (Figure 4B). Obviously, cells with suppressed eIF3a expression exhibited a reduced ability to form pseudopodia. In particular, filopodia, a special type of pseudopodia, are thin, finger-like exploratory protrusions that are indispensable for cell migration (Mattila and Lappalainen, 2008). Cells in the control group formed more filopodia protrusions with punctate actin and cortactin structures, whereas eIF3a downregulation explicitly hampered the occurrence of filopodia (Figure 4C). Altogether, these results suggested that pseudopodia formation may be a crucial mechanism involved in eIF3a-promoted tumor cell invasion.

### eIF3a Regulates Cytoskeletal Remodeling and Pseudopodia Formation *via* RhoA and Cdc42

**FIGURE 5:**
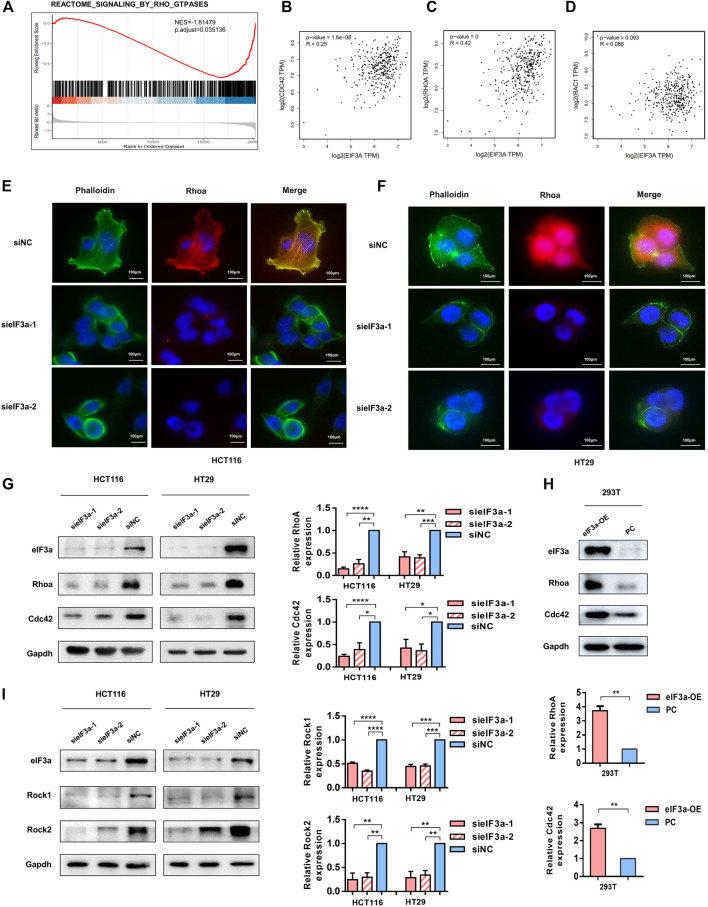
eIF3a affects tumor cell motility by activating Cdc42 and RhoA. **(A)** GSEA outcomes of eIF3a revealed enrichment in the Rho GTPase signaling pathway. **(B–D)** Correlation analyses between eIF3a and Cdc42 **(B)**, RhoA **(C)**, and Rac1 **(D)** were performed in GEPIA. **(E,F)** Immunofluorescence assays were performed in control and eIF3a-silenced cells. F-actin stained with FITC-phalloidin (green) and RhoA (red) are shown. **(G,H)** eIF3a was silenced in HCT116 and HT29 cells and upregulated in 293T cells. Western blot analyses were performed to evaluate Cdc42 and RhoA protein levels. **(I)** Western blot assays were used to detect alterations in Rock1 and Rock2 expression. sieIF3a, small interfering RNA targeting eIF3a; siNC, control small interfering RNA; eIF3a OE, eIF3a overexpression; PC, control plasmid.

In light of the observation that eIF3a regulates pseudopodia formation, we then investigated the underlying mechanism of this process. By performing gene set enrichment analysis (GSEA), we found that eIF3a was significantly associated with constitutive activation of the Rho GTPase signaling pathway **(** Figure 5A). Previous studies have demonstrated that Rho family proteins, especially RhoA, Rac1, and Cdc42, play key roles in cell invasion by regulating the assembly of actin stress fibers, the key process for pseudopodia formation (Hodge and Ridley, 2016; Arnold et al., 2017; Chang et al., 2019). In particular, our correlation analysis results indicated a positive correlation between the expression of eIF3a and RhoA and Cdc42 but not Rac1 (Figures 5B–D). Given that actin bundle formation is crucial for filopodia generation and RhoA plays a vital role in cytoskeleton reorganization, we conducted immunofluorescence experiments and investigated the status of RhoA and cytoskeleton formation using FITC-conjugated phalloidin in response to eIF3a suppression. Visualization of the actin cytoskeleton revealed a significant decrease in the formation and assembly of actin stress fibers after eIF3A knockdown. RhoA expression was also obviously inhibited (Figures 5E,F). We then decreased eIF3a expression in HCT116 and HT29 cells or overexpressed eIF3a in 293T cells to evaluate alterations in RhoA and Cdc42 protein levels. The results revealed a significant decrease in RhoA and Cdc42 expression after eIF3a knockdown (Figure 5G). However, the opposite trend was found in the overexpression group (Figure 5H). Rocks are the most typical downstream effectors of Rho and play irreplaceable roles in RhoA-induced stress fiber formation by phosphorylating several downstream targets, including LIM kinase (Limk), myosin light chain (MLC), and myosin phosphatase-targeting subunit 1 (Mypt1). Thus, we also evaluated the expression of two isoforms of mammalian Rocks, Rock1 and Rock2, to further verify the regulatory role of eIF3a in RhoA and actin cytoskeleton reconstruction (Figure 5I). According to the above results, we hypothesized that the regulatory effect of eIF3a on the invasive properties of colorectal cancer cells may be accomplished by RhoA and Cdc42 activation.

### eIF3a Regulates RhoA and Cdc42 *via* Translational Activation

**FIGURE 6:**
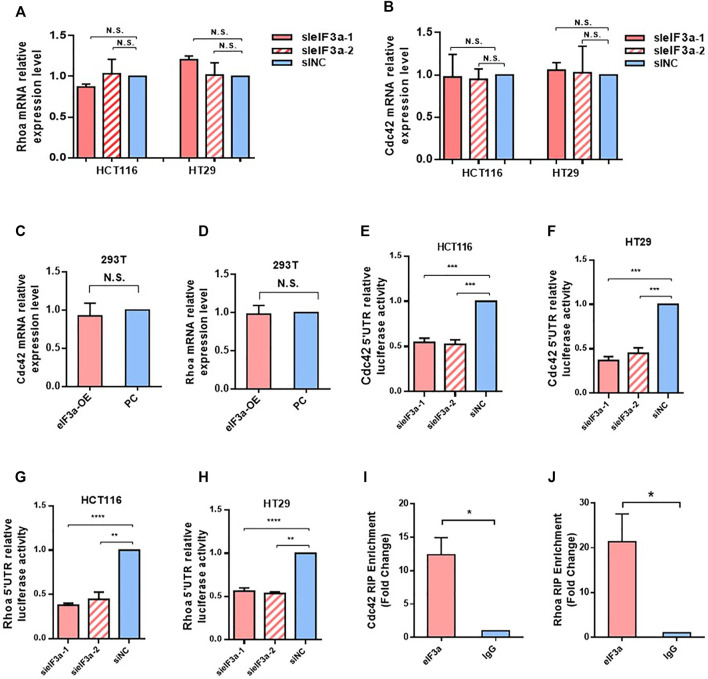
eIF3a regulates Cdc42 and RhoA *via* translational activation. **(A,B)** q-PCR assays were used to evaluate Cdc42 **(A)** and RhoA **(B)** mRNA expression. Statistical results were obtained from three independent experiments. **(C,D)** eIF3a was overexpressed in 293T cells, and q-PCR analysis was performed to measure Cdc42 **(C)** and RhoA **(D)** mRNA expression. **(E–H)** HCT116 and HT29 cells were treated with eIF3a and control siRNAs and then subjected to luciferase reporter gene assays. The translational activation ability of eIF3a on the 5′ UTRs of Cdc42 **(E,F)** and RhoA **(G,H)** was evaluated. **(I,J)** RIP assays were performed to verify the direct interaction among eIF3a and the 5′ UTR of Cdc42 **(I)** and RhoA **(J)**. sieIF3a, small interfering RNA targeting eIF3a; siNC, control small interfering RNA.

Given that eIF3a is widely known as a translation regulator and eIF3a expression led to no difference in Cdc42 and RhoA mRNA levels (Figures 6A–D), we hypothesized that the regulatory effect of eIF3a on Cdc42 and RhoA occurred during the translation process. To verify our hypothesis, we introduced the 5′ UTR regions of Cdc42 and RhoA into luciferase reporter gene vectors and tested the luciferase activities. As shown in Figures 6E–H, decreasing eIF3a significantly inhibited the translation initiation activity of the 5ʹ UTR regions of Cdc42 and RhoA. Finally, by performing RIP analysis, we proved that the eIF3a protein could directly bind to the mRNA of Cdc42 and RhoA (Figures 6I,J). Together, these results indicated that eIF3a regulates Cdc42 and RhoA expression *via* translational regulation.

### The Functional Verification of eIF3a in Clinical Tissues and Animal Model Tissues

**FIGURE 7:**
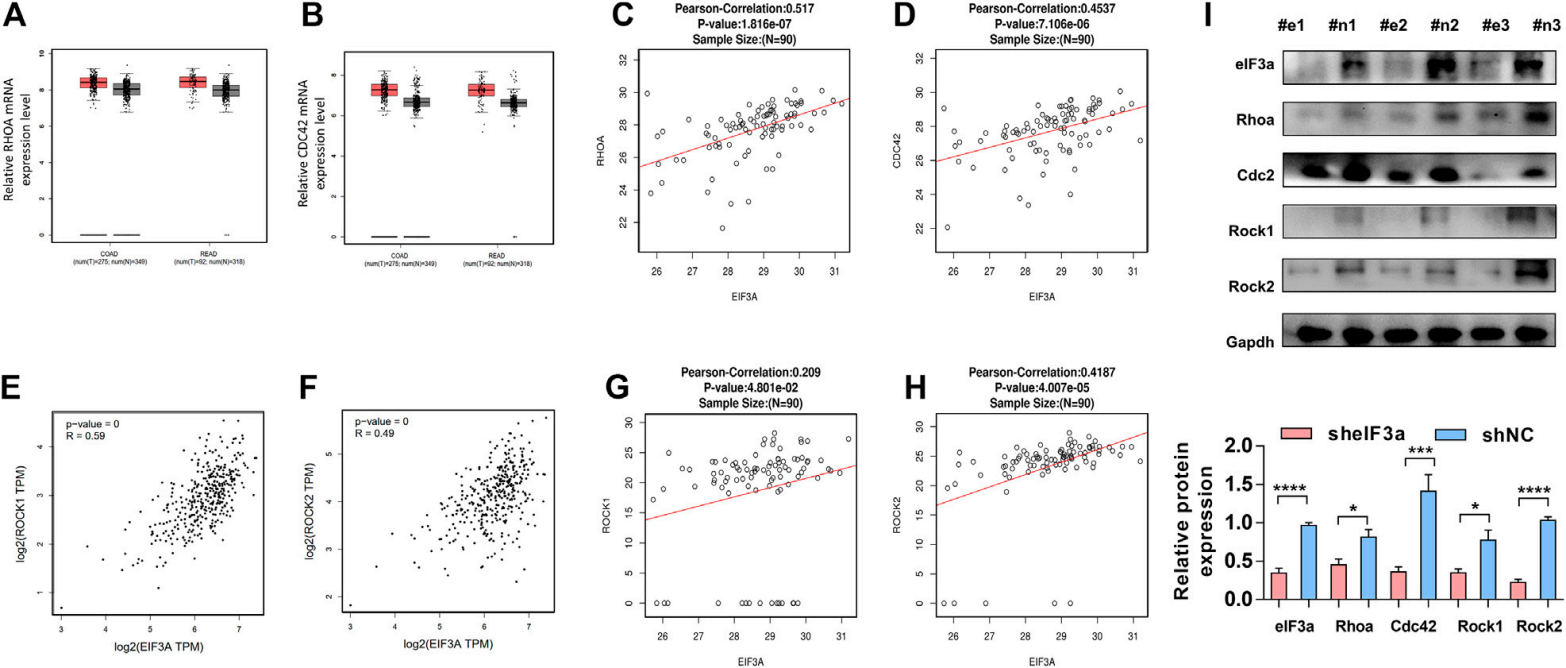
The functional verification of eIF3a in clinical tissues and animal model tissues **(A,B)**. The expression of RhoA and Cdc42 was evaluated in colorectal tumor tissues versus normal tissues. These assays were performed online by taking use of GEPIA database. **(C,D)** The protein expression of eIF3a, RhoA, and CDC42 was analyzed in tumor tissues of colorectal cancer patients, and correlation analyses were performed at LinkedOmics database. **(E–H)** The mRNA **(E,F)** and protein **(G,H)** expressions of eIF3a, Rock1, and Rock2 were analyzed in tumor tissues of colorectal cancer patients, and correlation analyses were performed at GEPIA and LinkedOmics database, respectively. **(I)** The protein samples were extracted from mouse tumor tissues, and western blotting was performed. #e1-3, tumors from mouse injected with sheIF3a HCT116 cells; #n1-3, tumors from mouse injected with shNC HCT116 cells.

To further confirm the regulatory function of eIF3a on RhoA, Cdc42, and their downstream genes, we verified the above observations in clinical colorectal tumor samples. Similar to eIF3a, RhoA and Cdc42 represent higher expression in colorectal tumor tissues compared to adjacent normal tissues (Figures 7A,B). The positive correlations between the protein expression of eIF3a and RhoA and Cdc42 were also confirmed (Figures 7C,D). The correlation analysis was conducted online at the LinkedOmics database (http://www.linkedomics.org/). Except these, the positive correlation between eIF3a and Rock1 and Rock2 were also verified at both mRNA (Figures 7E,F) and protein levels (Figures 7G,H). Finally, to study the regulatory role of eIF3a on RhoA, Cdc42, and their downstream genes *in vivo*, western blotting was used to test the expression of RhoA, Cdc42, and Rock1/2 in resected mouse tumor tissues described in Figure 3M (Figure 7I). These results further reveal the regulatory role of eIF3a in RhoA and Cdc42 in mouse and clinical patient tissues.

**FIGURE 8:**
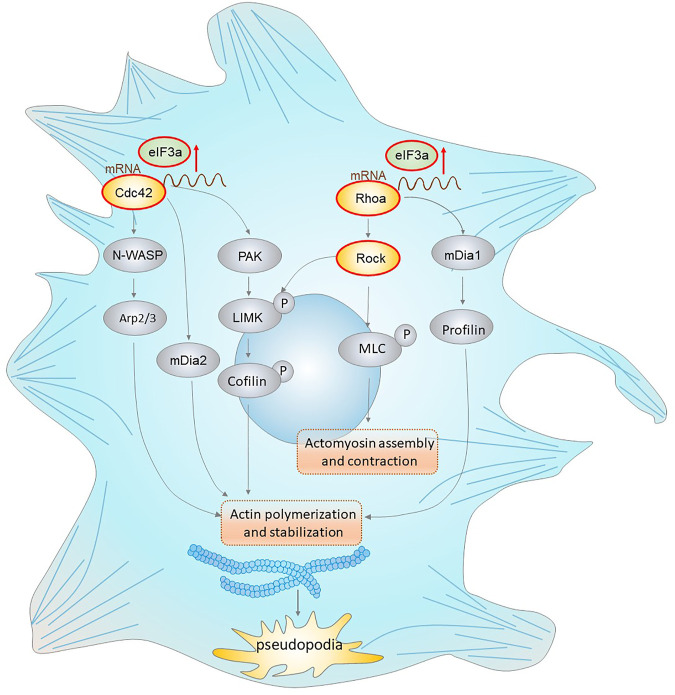
eIF3a regulates colorectal cancer metastasis by regulating RhoA/Cdc42 signaling at the translational level. This figure shows the translational regulatory role of eIF3a on Cdc42 and RhoA as well as their effectors that mediate actin cytoskeletal and pseudopodia formation. Cdc42 activates the Arp2/3 complex through WASP (Wiskott–Aldrich syndrome protein), leading to actin polymerization. Cdc42 also activates mDIA2 to promote the nucleation of unbranched actin filaments. Moreover, Cdc42 activates PAK family kinases, which further phosphorylate and activate LIMK (LIM domain kinase) and finally phosphorylate cofilin to inhibit its functions in actin filament severing and depolymerization. RhoA interacts with and activates ROCK, which further phosphorylates LIMK (LIM domain kinase) and activates cofilin. RhoA can also accelerate this process *via* the mDia1/Profilin axis. In addition, ROCK promotes MLC phosphorylation, which results in increased actomyosin assembly and contraction. eIF3a facilitates the above processes *via* translational regulation of Cdc42 and RhoA, which further promotes the acquisition of an invasive phenotype of malignant cells *via* actin cytoskeletal remodeling and pseudopodia formation.

In conclusion, our results demonstrated a novel role of eIF3a in the acquisition of tumor invasiveness. We also provide mechanistic insight into its translational activation ability on Cdc42 and RhoA, thus promoting pseudopodia formation and cytoskeleton reorganization of colorectal cancer cells (Figure 8). These findings highlight the therapeutic potential of eIF3a to suppress colorectal cancer metastasis.

## Discussion

To date, distant metastasis remains the dominant lethal cause of colorectal cancer, and no therapy effectively prevents disease progression (van der Pool et al., 2012; Hackl et al., 2014; Elferink et al., 2015; Rahbari et al., 2019). The role of eIF3a in cell migration has been studied in pancreatic ductal adenocarcinoma and hepatocarcinoma *in vitro* by conducting Transwell and wound healing assays. The results showed that eIF3a downregulation inhibited the motility of both cell lines (Wang et al., 2016; Chen et al., 2020). However, no detailed investigations have been conducted to explain the specific mechanisms involved in this process.

This study comprehensively investigated the role and mechanism of eIF3a in tumor invasion and metastasis in colorectal cancer. Given the proto-oncogenic role of eIF3a in several other types of tumors, we investigated the specific features of eIF3a in colorectal cancer. We first evaluated its expression in a panel of clinical samples and discovered notably differential eIF3a expression in colorectal tumors compared with normal tissues at both the mRNA and protein levels. Additionally, primary tumors exhibiting higher eIF3a expression are more likely to develop metastatic disease. Survival analysis indicated the promising efficiency of eIF3a expression in predicting the prognosis of colorectal patients. Furthermore, knockdown of eIF3a in colorectal cancer cell lines significantly impaired cellular proliferation, and both findings were verified *in vitro* and *in vivo*. The above results supported our hypothesis that eIF3a promotes tumor cell proliferation, which is consistent with the findings in previous studies. It has been reported that downregulation of eIF3a reduces the cell proliferation rate by prolonging the cell cycle rather than changing the cell cycle distribution (Dong et al., 2009). Mechanistically, eIF3a inhibits the translation of p27^kip1^, a cyclin-dependent kinase (CDK) inhibitor that regulates cell cycle progression at G1 phase (Dong and Zhang, 2003). In addition, eIF3a also translationally upregulates RRM2, which controls malignant cell cycle progression and proliferation by regulating DNA synthesis (Dong et al., 2004). We also confirmed these findings in colorectal cancer cell lines.

Tumor cell spread and metastasis to distant locations require the acquisition of an invasive phenotype (Chaffer et al., 2016). The migration of tumor cells is a complex and dynamic process with continuous reconstruction of cellular architecture (Suhail et al., 2019). To actively migrate, cells first undergo *de novo* actin polymerization and develop clearly defined polarity. This step is followed by cytoskeletal rearrangement and the formation of membrane protrusions (Gupton and Waterman-Storer, 2006). Rho proteins are the main regulators of this process by controlling cellular actin and microtubule dynamics (Jansen et al., 2018). Cdc42 possesses a crucial role in filopodia formation. This process is accomplished primarily through actin filaments by arranging in bundles and forming an actin core. RhoA is involved in stress fiber formation and focal adhesions, and its activation leads to bundling of existing actin filaments into stress fibers and promotes myosin to generate contractile forces (Chan et al., 2010; Worzfeld et al., 2012; Bon et al., 2016). The detailed mechanism is summarized in Figure 7.

Based on GSEA, we found that eIF3a expression was significantly correlated with the Rho GTPase signaling pathway, which represents a novel explanation of the proinvasive role of eIF3a. With further research, we found that eIF3a suppression notably inhibited pseudopodia formation and cytoskeleton reorganization, both of which are essential properties necessary for tumor cell invasiveness. Mechanistically, this regulatory effect is realized through the translational activation of Cdc42 and RhoA, and this conclusion is based on their differential expression at the protein level instead of at the mRNA level after eIF3a knockdown or overexpression together with outcomes in the luciferase reporter assay. The direct contact of eIF3a with Cdc42 and RhoA was further detected by RIP analysis. To further confirm the regulatory effect of eIF3a on RhoA, the expression of Rock1 and Rock2, two Rock subtypes, was tested. Rocks are important Ser/Thr kinases downstream of RhoA and promote RhoA-induced stress fiber formation and focal adhesion by phosphorylating several downstream targets, such as LIM kinase, MLC, and MYPT1. Rock has been considered an antitumor target, the inhibition of which suppresses metastasis in a variety of types of cancer (Tang et al., 2008; Grise et al., 2009; Matsuoka and Yashiro, 2014). Rock1 and Rock2 expression was consistently reduced after eIF3a suppression, which further confirms the regulatory ability of eIF3a in RhoA-induced stress fiber formation.

To summarize, our work demonstrated the requirement of eIF3a in the translational activation of Cdc42 and RhoA, which further contributes to pseudopodia formation and cytoskeleton reorganization. Through this mechanism, malignant cells successfully acquire and transform into a more aggressive and invasive phenotype and finally spread to distant organs. Our results support the conclusion that eIF3a is a potential biomarker for colorectal cancer progression and metastasis. We also provided a potential therapeutic target that may be used to effectively control tumor metastasis and progression.

## Data Availability

The original contributions presented in the study are included in the article/Supplementary Material, further inquiries can be directed to the corresponding authors.

## Supplementary Material

Supplementary Figure S1 Upregulation of eIF3a promotes 293T cell proliferation and migration. **(A)** eIF3a was overexpressed using a specific plasmid in the 293T cell line. Representative images (left) and quantification (right) of wound healing assays are presented. Images were captured at 0, 24, and 48 h. **(B)** The viability of 293T cells was analyzed by CCK-8 assay. **(C)** Transwell penetration assay. Typical images and quantification of invading cells are presented. eIF3a-OE, eIF3a overexpression; PC, control plasmid.

Supplementary Figure S2 eIF3a contributes to cell proliferation by regulating RRM2 and p27^kip1^. **(A)** HCT116 and HT29 cells were treated with eIF3a and control siRNAs followed by western blot assays.

## Abbreviations

Cdc42, cell division cycle 42; Rho family GTPases, Rho family of small guanosine triphosphatases; RhoA, ras homolog family member A; Rock, Rho-associated serine-threonine kinase; eIF3a, eukaryotic translation initiation factor 3a.
