# Supplementary figures and images for: eIF3a Regulates Colorectal Cancer Metastasis via Translational Activation of RhoA and Cdc42
Source: Front Cell Dev Biol. 2022 Mar 1;10:794329. doi: 10.3389/fcell.2022.794329 (PMC8921074; doi:10.3389/fcell.2022.794329)

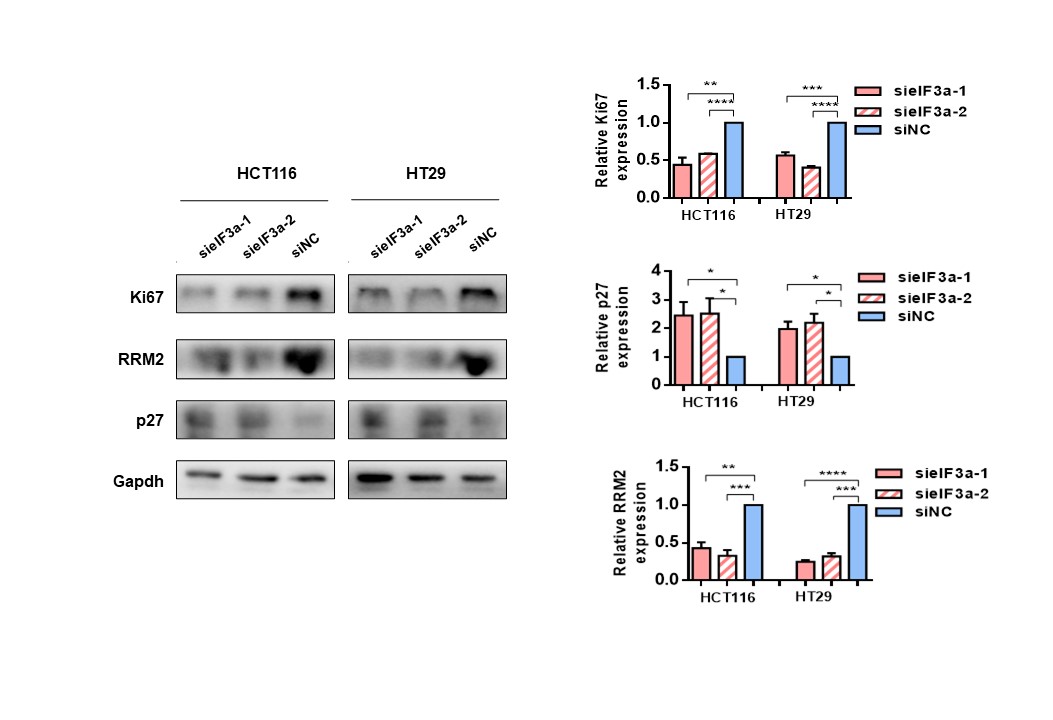

Supplement: Supplementary file 1 [file Image2.JPEG]

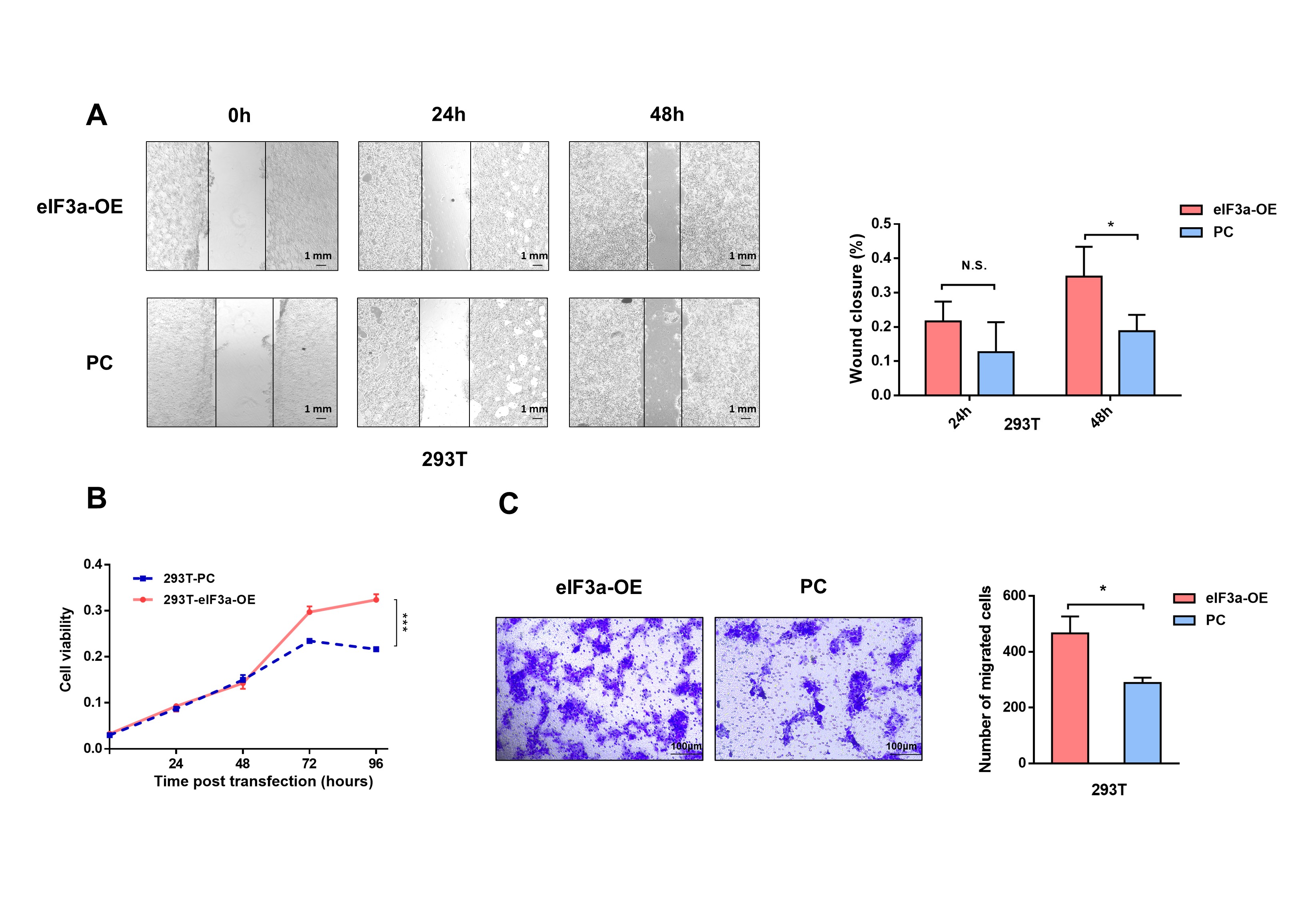

Supplement: Supplementary file 2 [file Image1.jpg]
