# Supplementary material for: eIF3a Regulates Colorectal Cancer Metastasis via Translational Activation of RhoA and Cdc42
Source: Front Cell Dev Biol. 2022 Mar 1;10:794329. doi: 10.3389/fcell.2022.794329 (PMC8921074; doi:10.3389/fcell.2022.794329)
Supplement: Supplementary file 3 [file DataSheet1.docx]

| **Supplementary Table 1. siRNA sequences of candidate genes for knockdown** | |
| --- | --- |
| **Gene** | **Sequences** |
| eIF3a | si1: 5'-CGTGCTGATGATGATCGGTTT-3' |
|  | si2: 5'-GCGCCTTGAGAGTCTGAATAT-3' |

| **Supplementary Table 2. Primers of candidate genes for RT-PCR** | | |
| --- | --- | --- |
| **Gene** | **Sequences** | |
| eIF3a | | F: 5'-TCAAGTCGCCGGGACGATA -3' |
|  | | R: 5'-CCTGTCATCAGCACGTCTCCA-3' |
| Rhoa | | F:5'- GATTGGCGCTTTTGGGTACAT -3' |
|  | | R:5'- AGCAGCTCTCGTAGCCATTTC -3' |
| Cdc42 | | F: 5'- CCATCGGAATATGTACCGACTG -3' |
|  | | R: 5'- CTCAGCGGTCGTAATCTGTCA -3' |
| Gapdh | | F: 5'-ACAGCCTCAAGATCATCAGC -3' |
|  | | R: 5'-GGTCATGAGTCCTTCCACGAT-3' |

| **Supplementary Table 3. shRNA sequences of candidate genes for knockdown** | |
| --- | --- |
| **Gene** | **Sequences** |
| eIF3a | sh1: CGTGCTGATGATGATCGGTTT |
